# PERSONALITY PROFILES AND LIFE SATISFACTION IN AGING: A LATENT GROWTH CURVE MODELING APPROACH

**DOI:** 10.1093/geroni/igaf122.1764a

**Published:** 2025-12-31

**Authors:** Yeon Ji Ryou, Momoho Kakuta

**Affiliations:** Iowa State University, Ames, Iowa, United States; The University of Osaka, Suita, Osaka, Japan

## Abstract

This study examined life satisfaction trajectories among individuals 65 years and older using growth mixture modeling (GMM) with pre-identified personality profiles from latent profile analysis (LPA) and covariates. The targeted sample (N = 2,424) was from the merged data of the 2020 RAND Health and Retirement Study (HRS) and 2022 Core data, covering three time points (T1=2012/2014, T2=2016/2018, T3=2020/2022). Given the alternating design of data collection, each point pooled two consecutive waves for the complete sample for psychosocial questions. Three personality profiles: Resilient Extraverts (53.26%), Balanced (40.18%), and Sensitive Introverts (6.56%), were identified and used to predict life satisfaction trajectories. Results found significant baseline differences across the sub-groups. Resilient Extraverts showed the highest life satisfaction at T1 (B = 1.984, SE = .44, p < .001), followed by the Balanced group (B = 1.695, SE = .44, p < .001) and Sensitive Introverts (B = 1.405, SE = .44, p < .01). Despite these initial differences, all groups demonstrated an increasing trend in life satisfaction over time (p < .01). Better subjective health, older age, and being married were positively associated with life satisfaction at baseline. However, older age was linked to a slower rate of change. These findings suggest that psychosocial resources could involve sustaining well-being. Although aging was associated with a slower rate of growth, the overall upward trend suggests the possibility of continued psychological well-being, emphasizing the need for targeted interventions that utilize individual personality strengths and social resources to enhance well-being in later life.

